# The “Woggle” Technique for Venous Access Site Management: An Old Technique for a New Need

**DOI:** 10.3390/jcm12186087

**Published:** 2023-09-20

**Authors:** Gabriele Tumminello, Lucia Barbieri, Carlo Avallone, Nello Bellissimo, Luca Mircoli, Federico Colombo, Marco Vicenzi, Massimiliano Ruscica, Stefano Carugo

**Affiliations:** 1 Department of Cardio-Thoracic-Vascular Diseases, Foundation IRCCS Ca’ Granda Ospedale Maggiore Policlinico, 20154 Milan, Italy; lb.luciabarbieri@gmail.com (L.B.); luca.mircoli@policlinico.mi.it (L.M.); federico.colombo@policlinico.mi.it (F.C.); marco.vicenzi@unimi.it (M.V.); massimiliano.ruscica@unimi.it (M.R.); stefano.carugo@unimi.it (S.C.); 2 Dyspnea Lab, Department of Clinical Sciences and Community Health, University of Milan, 20122 Milan, Italy; carloavallone95@gmail.com (C.A.); nellomanuel.bellissimo@studenti.unimi.it (N.B.); 3 Department of Pharmacological and Biomolecular Sciences “Rodolfo Paoletti”, Università degli Studi di Milano, 20133 Milan, Italy

**Keywords:** percutaneous structural heart intervention, large bore venous access, percutaneous closure vascular systems

## Abstract

Background: Several closure devices are routinely used for percutaneous arterial access, while a relatively low number is available for the management of large bore venous accesses. The Woggle technique is a modification of the purse-string suture which was introduced several years ago in patients undergoing hemodialysis. Methods: A population of 45 patients who underwent transvenous femoral structural heart interventions was retrospectively evaluated. The Woggle technique consists of a purge string suture with a collar to maintain the tension as stable over time and a suture lock to tighten the suture. Results: Sheaths magnitude ranged from 8 French (F) to 14 F. A rapid post-procedural hemostasis was achieved in the whole population, and in 95% of cases, definite hemostasis was obtained after the first single release; the mean time of release was 302 ± 83 min. Although no relevant bleedings were reported, a significant reduction in hemoglobin levels was found in the whole population. This decrement was statistically significant only in the group with sheaths higher than 12 F. A single mild local hematoma was recorded in the group in which smaller sheaths were used. Seventy-two percent of patients were pre-treated with a dual antiplatelet therapy. Conclusions: The Woggle technique has shown to be a simple, effective, and safe approach for the management of large bore venous in percutaneous structural heart interventions.

## 1. Introduction

Vascular access sites for percutaneous structural heart interventions can be either venous or arterial. Several closure devices are routinely used for arterial access [1], while a relatively low number is available for the correct management of venous approaches. Achieving hemostasis in venous access is similar to arterial district and can be achieved by different approaches (e.g., manual compression, mechanical external compression, and dedicated devices). However, only in the case of venous access, some studies reported purse-string suture as a valid alternative even in large bore [2]. Although very effective, the suture can be difficult to remove if the knot becomes buried in tissues narrowing the puncture sites, which may be puckered. Furthermore, once cut, the purse-string suture can not be re-used if final hemostasis is not achieved. This leads to a complication rate of up to 3.1% [3]. Within this context, there is a need for a technique that allows both the compression to be adjusted over time and the points to be easily removed. This is required to obtain an effective compression and to adjust it over time with little effort and in a simple way [4]. All these aspects are enclosed in the Woggle technique, which is based on circumferential tension at the puncture site without torsion or traction. The suture can be tightened or loosened as necessary (i.e., adjusting the amount of force needed to achieve hemostasis). Although the Woggle technique has been used to achieve hemostasis in the hemodialysis arterio-venous fistula catheterization sites, its effectiveness is unknown in interventional cardiology percutaneous large bore venous access sites [4,5]. Previous studies, in the field of hemodialysis, found that no patients developed aneurysms or pseudoaneurysms at the puncture site after the use of a purse-string suture and a suture lock device; in addition, most puncture sites had no significant changes in access diameter [6]. The guidelines of the Society of Interventional Radiology (SIR) reported no major complications, but some minor ones that required simple management during the procedure of hemodialysis access without post-procedural consequences or additional therapy occurred (e.g., venous rupture and graft rupture, which could be managed without surgery or blood transfusion) [7].

Thus, the aim of the present study was to evaluate either the safety of the Woggle technique or the feasibility of the management of venous access sites used for percutaneous structural heart interventions which are mostly represented by left atrial appendage occlusion (LAAO) and patent foramen ovale (PFO) closure.

## 2. Materials and Methods

We performed a retrospective cohort study of patients who underwent percutaneous structural heart interventions, LAAO and PFO closure, needing venous access sites with a large bore. Data were collected from May 2020 and May 2023 at San Carlo Borromeo Hospital and Fondazione IRCCS Ca’ Granda Ospedale Maggiore Policlinico, Milan, Italy. From May 2020, we routinely used the Woggle technique, and almost all patients treated with trans venous percutaneous structural heart intervention were included. A large bore was defined as when the procedure needed a sheath over 8 French (F). All demographic and clinical data were prospectively collected in a dedicated database. The study was conducted according to good clinical practice, institutional guidelines, national legal requirements, European standards, and the revised Declaration of Helsinki. All patients provided informed consent for the use of personal data. Hypertension was defined as a previous diagnosis of arterial hypertension under optimal treatment or measurement of systolic blood pressure at rest (BP) >140 mmHg and/or a diastolic BP >90 mmHg. The diagnosis of diabetes was based on a previous history of diabetes treatment, fasting glycemia >126 mg/dL or glycosylated hemoglobin >6.5%. Body mass index was calculated as weight/height (Kg/m^2^). Hypercholesterolemia was defined as a previous history of hypercholesterolemia, chronic treatment with any lipid-lowering drug (e.g., statins) [8] at admission, or fasting total cholesterol >200 mg/dL. Creatinine clearance was assessed using the CKD-EPI formula [9]. Bleeding events were defined according to the criteria of the Bleeding Academic Research Consortium (BARC) [10]. Local hematoma at the site of catheterization was defined as a localized swelling with an area of clotted blood changing the color of the skin, but with no evidence of active bleeding.

### 2.1. Technique

**Figure 1:**
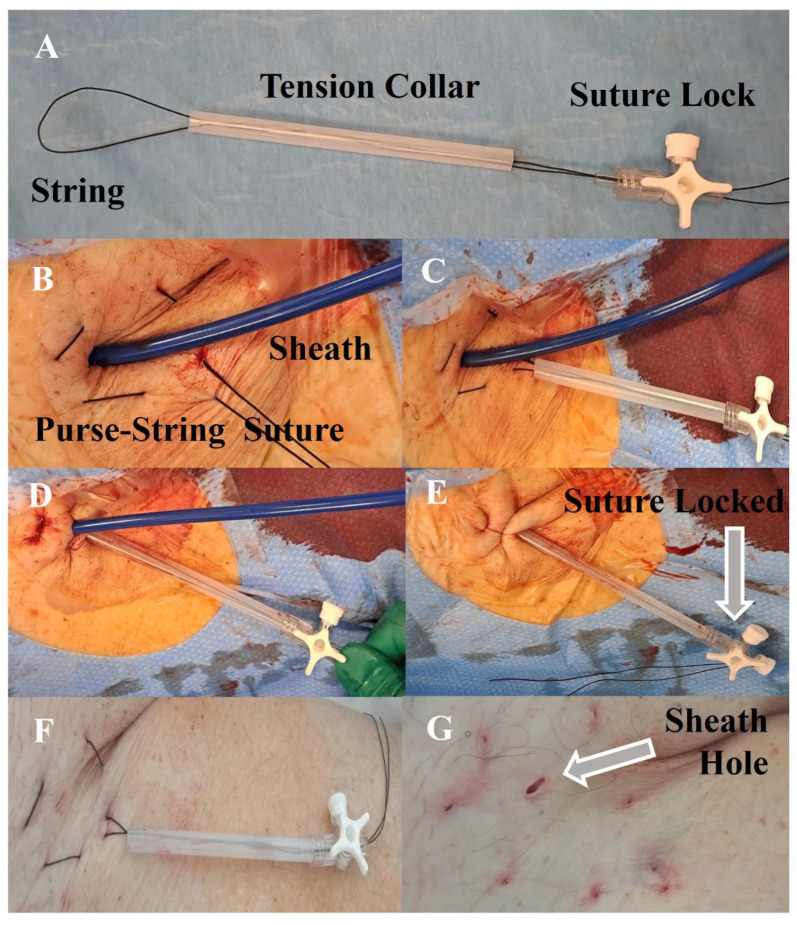
Woggle technique components and application. (**A**) Components of Woggle system; (**B**) purse-string suture around sheath; (**C**) set up of the Woggle system; (**D**) tensioning the system while removing sheath; (**E**) system locked with immediate hemostasis; (**F**) system de-tensioned (re-tensionable if needed) for definite hemostasis; (**G**) removal.

The Woggle technique is a modification of the purse-string suture (Figure 1). The purse-string is performed around the sheath introduced in the venous access site. The purse-string suture is a continuous stitch passed through the superficial skin layers to deep layers and back to the superficial layers again at 4 or 5 points around the sheath with the two ends of the string free. This makes a constricting thread like a “bag mouth” around the sheath. A tension collar (plastic introducer or similar device) is placed over the ends. The collar maintains the tension of the string as stable over time as an elastic component of the mechanism. The sheath is removed, and the purse-string suture is tightened until hemostasis is achieved. The ends of the string are secured with a suture lock (hemostat or stopcock) to tighten the suture without making a knot. The device is left in place for the required time to achieve hemostasis, which is influenced by several factors (e.g., the coagulative state of the patients, the dimension of the sheath and antiplatelets or anticoagulant therapies). During this time, the purse-string suture may be tightened or de-tensioned according to the need, pulling or releasing the ends of the wire and locking again with the stopcock. When hemostasis is achieved, the Woggle device is released, and the suture is removed. This technique allows the patient to be discharged without a suture in place.

### 2.2. Statistical Analysis

Statistical analysis was performed with the IBM SPSS Statistic Release v28. Continuous variables were tested for normal distribution with the Shapiro–Wilk test and expressed as mean ± standard deviation if normally distributed, or median and interquartile ranges if not. Categorical variables were expressed as absolute and as percentage frequencies, and differences were tested using the chi-squared test. A Mann–Whitney test was used for non-normal quantitative variables, while Student’s *t*-test was used to compare differences between means. The statistical significance of *p*-values was set at 0.05 and the confidence intervals (CI) at 95%.

## 3. Results

A total of 45 patients were recruited and all were treated with the Woggle technique for large-bore venous closure. According to the total of the procedures, 33% were LAAO and 66% were PFO closures. LAAO was performed in patients who were affected by chronic atrial fibrillation and with a high risk of bleeding, whereas PFO closures were performed in patients affected by ischaemic stroke [11,12]. The clinical characteristics of the whole population are fully described in Table 1. Patients were more often male (62%) with a mean age of 57.6 ± 16.5 years. Hypertension was reported in 46% and the mean BMI was 20.9 ± 3.8 kg/m^2^. Five patients (11%) were on hemodialysis, and only one had a history of previous hemorrhage. In all patients, procedures were performed through venous femoral access with sheath magnitudes ranging from 8 to 14 F, and 20 patients (44%) had a size larger than 12 F. Unfractionated heparin was infused during all procedures, with a mean cumulative dose of 92 ± 22 UI/kg; protamin to reverse heparin was never administered at end of the procedures. At the time of intervention, 40 patients (89%) were on therapy with aspirin and 26 (58%) were on dual antiplatelet therapy (DAPT) with clopidogrel. The mean procedural time was 65 ± 35 min with a success rate of 100%. The Woggle technique was successful in the achievement of immediate post-procedural hemostasis for the whole population and, in 95% of cases (43/45 patients), definite hemostasis was achieved after the first single release that was performed at a mean time of 302 ± 83 min after the procedure. The time of first release was decided by the first operator based on the heparin’s total half-life and the current antiplatelet therapy. In the two patients without definite hemostasis, the first release was sufficient to re-stretch the suture and lock it again with the suture lock. A second attempt was performed after roughly 30 min. Technical achievement for hemostasis, defined as no supplementary requirement for further manual compression, manipulation, or protracted examination, was accomplished in 100% of patients. There were no differences in creatinine before and after the procedure, while a significant difference in hemoglobin value was found (12.8 ± 2.2 vs. 12.4 ± 2.1 gr/dL, *p* = 0.01, Table 2). No major access complications or no BARC bleeding greater than 2 were reported. When the population was stratified into two groups according to the sheath magnitude (8–11 F and 12–14 F sheath), a single patient, assigned to the 8–11 F group, experienced local hematoma (Table 3). In this group, DAPT was most represented (Table 3). Patients in the 12–14 F sheath group had a greater drop in the hemoglobin values compared with patients treated with smaller sizes (Table 4). At discharge, 36 patients (80%) were on DAPT, 4 patients (8.8%) were on single antiplatelet therapy with acetylsalicylic acid, 1 patient was anticoagulated with a direct oral anticoagulant and 4 (8.8%) patients received any antithrombotic treatment due to the hemorrhagic risk. There were no thromboses, false aneurysms or access site infections that could be linked to the technique, and no reported cases of delayed bleeding after the discharge.

## 4. Discussion

The main findings of the present study are the safety and efficacy of the Woggle technique to achieve hemostasis after percutaneous venous large bore procedures for LAAO and PFO closure. Indeed, to the best of our knowledge, few studies have evaluated the Woggle technique to achieve hemostasis mostly in the hemodialysis arterio-venous fistula [4,5]. The recent increase in the type and number of structural transvenous heart interventions brought a new need: a simple, safe, and feasible system for venous hemostasis at the end of procedures. The risk of complications is increased with the development of cardiac interventions that require large-bore sheaths. Furthermore, interventions are carried out in older patients and patients at a high risk of bleeding treated with strong antiaggregating drugs, such as LAAO patients. Access site complications may affect the patient’s discomfort, immobilization, duration of hospitalization and, in the end, prognosis [13]. Thus, the management of vascular site access, even transvenous, is very important: an extended manual compression is routinely used, although it is time-consuming and may be very uncomfortable for patients. In addition, the use of a vascular device is expensive and not free of complications [3]. A recent paper investigated the use of the simple purse-string suture after MitraClip intervention, which proved to be feasible and safe [2]. Compared to a standard purse-string suture, the Woggle technique allows controlling and modulating the tension of the system over time. A further advantage of this technique lies in the possibility of tightening or loosening the suture several times, tailoring the hemostasis process to a single case. At the time of supposed hemostasis, the system is allowed to be completely de-tensioned, but only partially released, in order to maintain the possibility of re-tightening in case of bleeding. Conversely, the simple purse-string suture needs to be cut to be released, thus losing the possibility of being re-used in the case of persistent bleeding. Due to this limitation, the purse-string suture needs to be released when the hemostasis is almost obtained. After the Mitraclip procedure, the purse string is released after 24 h, limiting the mobilization of the patients. In our population, definite hemostasis was achieved in 95% of cases at the first release performed at a mean time of 302 +/− 83 min after the end of the procedure. This result was possible because operators were confident to release the Woggle as soon as possible. In case hemostasis was not obtained, the system was re-tensioned effectively and some miutes were allowed to pass. Due to its continuous mouldability, the Woggle technique is simpler compared to the purse-string suture. Because the purse-string suture is left in place for 24 h, it is recommended to perform an EchoDoppler evaluation after the removal to rule out vessel occlusions or deep vein thrombosis [2]. One of the reasons we did not register any complication may be that the Woggle system can be left in place for less time than a standard purse-string suture. Only one mild local hematoma was recorded in our population in the group of smaller sheaths. In this group, the majority of patients (72%) were already on DAPT before the procedure. In the group of bigger sheaths, composed mostly of LAAO patients, with a higher intrinsic bleeding risk and a significant minor administration of DAPT before the procedure with respect to the smaller sheath group (Table 3), we did not register any hematoma. This suggests that the development of hematoma may be related more to the antiplatelet/anticoagulation state of the patients than to the application of the technique, which is more influenced by the dimension of the sheaths itself. We did not register any clinically relevant bleeding, but we did register a significant reduction in hemoglobin levels after the procedure in the whole population. Dividing the population into two groups according to the dimension of the sheaths, the loss in hemoglobin was significant only among the 12–14 F group (Table 4). This may be explained by major bleeding during the procedure secondary to bleeding caused by the sheaths, and the consequent bigger delivery systems, rather than failure of the hemostasis technique after the procedure. Moreover, in our centers, the LAAO patients routinely underwent consistent hydration before the procedure to obtain the optimal left atrial distension, which is mandatory for correct device implantation. Therefore, this may influence the hemoglobin hematic concentration with a consequent reduction in the final hemoglobin value. In our population, only a single case of transfusion was reported after the procedure; however, this was required for a patient who underwent hemodialysis with known chronic anemia and did not manifest any hematoma or bleeding. The learning curve was irrelevant because all the operators in our center used the simple purse-string suture. Finally, the Woggle technique is cost-effective. A mechanized system, like Proglide, is hypothetically more expensive compared to a monofil silk suture and a stopcock. In our experience, venous hemostasis with the Woggle technique can be accomplished without any chronic scars, as previously described after percutaneous skin closure using a purse-string suture for 15 F sheaths [14].

## 5. Conclusions

The Woggle technique is a simple, effective, and safe method for the management of large-bore venous percutaneous structural heart interventions.

## Figures and Tables

**Table 1:** Population and procedural characteristics.

Clinical Characteristics	(n = 45)
Age	57.6 ± 16.5
Male sex (%)	28 (62)
BMI (Kg/m^2^)	20.9 ± 3.8
Hypertension (%)	21 (46)
Active smokers (%)	4 (9)
Hypercholesterolemia (%)	16 (35)
Diabetes (%)	6 (13)
Family history of CAD (%)	6 (13)
Previous stroke (%)	31 (69)
Atrial fibrillation (%)	14 (31)
Dialysis (%)	5 (11)
Previous hemorrhage (%)	1 (10)
Left ventricular ejection fraction (%)	58 ± 8
**Procedural characteristics**	
LAAO (%)	15 (33)
PFO-c (%)	30 (66)
Success rate (%)	45 (100)
ASA during procedure (%)	40 (89)
DAPT during procedure (%)	26 (58)
French sheath	11.6 ± 1.2
French sheath 12–14 (%)	20 (44)
Procedural time (min)	63 ± 35
Heparin (UI/Kg)	92 ± 22
**Woggle technique**	
Number of releases	1.04 ± 0.20
Single release (%)	43 (95)
Final time (minutes)	302–83
**Therapy discharge**	
No antithrombotic therapy	4 (8.8)
SAPT (%)	4 (8.8)
DAPT (%)	36 (80)
DOAC (%)	1 (2.2)
**In-hospital outcome**	
Days of hospitalization after procedure	2 ± 1.4
In hospital MACE (%)	0 (0)
In-hospital all-cause death (%)	0 (0)
Bleeding (BARC > 2) (%)	0 (0)
Bleeding requiring action (%)	0 (0)
Transfusion (%)	1 (2)
Local hematoma (%)	1 (2)

BMI = body mass index; CAD = coronary artery disease; LAAO = left atrial appendage occlusion; PFO-c = patent foramen ovale closure; ASA = acetylsalicylic acid; SAPT = single antiplatelet therapy; DAPT = dual antiplatelet therapy; DOAC = direct oral anticoagulants; MACE = major adverse cardiac events; BARC = Bleeding Academic Research Consortium. Data are expressed as mean ± SD or percentage.

**Table 2:** Hemoglobin and serum creatinine levels before and after the Woggle procedure.

Biochemistry	Pre Procedure	Post Procedure	*p*-Value
Hemoglobin (g/dL)	12.8 ± 2.2	12.4 ± 2.1	0.01
Serum creatinine (mg/dL)	1.47 ± 1.69	1.69 ± 2.17	ns

Data are expressed as mean ± standard deviation. ns, non-significant.

**Table 3:** Hematoma, DAPT therapy and hemoglobin pre and post-intervention according to different sheath dimensions.

Sheath	8–11 F	12–14 F	*p*-Value
n	25	20	
Hematoma (%)	1 (4)	0	ns
DAPT during procedure (%)	18 (72)	8 (40)	0.03
Delta hemoglobin (g/dL)	−0.4 [0.8]	−0.55 [1.0]	ns

DAPT = dual antiplatelet therapy; F, French. Data are expressed median [interquartile range]. n = numerosity.

**Table 4:** Hemoglobin levels before and after the procedure according to different sheath dimensions.

Clinical Characteristics	Pre Procedure	Post Procedure	*p*-Value
Sheath 8–11 hemoglobin	13.8 [3.05]	13.6 [3.0]	ns
Sheath 12–14 hemoglobin	12.6 [4.75]	11.7 [4.4]	0.039

Data are expressed median [interquartile range]. ns, non-significant.

## Data Availability

Data will be available on request to the corresponding author.
